# Comparative analysis of the mechanical limits of resistance in implant/abutment set of a new implant design: An *in vitro* study

**DOI:** 10.1371/journal.pone.0280684

**Published:** 2023-01-20

**Authors:** Marco Aurélio Bianchini, Nilton De Bortoli Junior, Berenice Anina Dedavid, Piedad N. De Aza, Sergio Alexandre Gehrke

**Affiliations:** 1 Post-Graduate Program in Implant Dentistry (PPGO), Federal University of Santa Catarina (UFSC), Florianópolis, Brazil; 2 Post-Graduate Program in Implant Dentistry, Paulista University, São Paulo, Brazil; 3 Department of Materials Engineering, Pontificial Catholic University of Rio Grande do Sul, Porto Alegre, Brazil; 4 Instituto de Bioingenieria, Universidad Miguel Hernández, Elche (Alicante), Spain; 5 Department of Biotechnology, Universidad Católica de Murcia (UCAM), Murcia, Spain; University of Vigo, SPAIN

## Abstract

**Objective:**

The aim of the present *in vitro* study was to evaluate the resistance on quasi-static forces and in the fatigue mechanical cycling of a new implant design compared to two other conventional implant designs.

**Materials and methods:**

Eighty-eight implants with their respective abutments were tested and distributed into four groups (n = 22 per group): Morse taper connection implant (MT group), conventional external hexagon implant (EH con group), new Collo implant of external hexagon with the smooth portion out of the bone insertion (EH out group), and new Collo implant of external hexagon with the implant platform inserted to the bone level (EH bl group). All the sets were subjected to quasi-static loading in a universal testing machine, and we measured the maximum resistance force supported by each sample. Another 12 samples from each group were submitted to the cyclic fatigue test at 4 intensities of forces (n = 3 per force): 80%, 60%, 40%, and 20%. The number of cycles supported by each sample at each force intensity was evaluated.

**Results:**

The three groups of implants with external hexagon connection had similar maximum strength values of the sets (*p* > 0.05). Meanwhile, samples from the MT group showed the highest resistance values in comparison to the other three groups (*p* < 0.05). In the fatigue test, the Collo out group supported a smaller number of cycles that led to the fracture than the other 3 groups proposed at loads of 80%, 60%, and 40%, and only at the load value of 20% all groups had the same performance.

**Conclusions:**

Within the limitations of the present *in vitro* study, the results showed that the new Collo implant performs better when installed at bone level.

## Introduction

Rehabilitative treatments for the replacement of missing teeth through osseointegrated implants have become routine practice within current dentistry, mainly due to their predictability and high degree of reliability achieved. However, development and research of new implant designs continue to be part of a search for excellence in long-term results and for ways to reduce possible problems related to this type of treatment. Among these problems, the most frequently encountered are related to the maintenance of the health of peri-implant tissues in the long term [1–3].

The stability of peri-implant tissues over time is directly linked to your quality, which, in turn, depends on the amount (volume) of hard and soft tissues present [4]. Normally, areas with a phenotype of thin tissue (hard and/or soft tissues), which have not been adequately healed to receive the implants, may be more susceptible to peri-implant problems, which can range from mucositis to peri-implantitis [5]. Several authors have related the amount (volume) of tissue around the cervical area of the implants with the possibility of some loss of support, disease in this area, and/or aesthetic problems [4–6].

In this sense, in an attempt to increase the volume of peri-implant tissues in the cervical area, numerous implant designs are present in the global market, each one with its characteristics and concepts seeking some benefit for patients. Regarding this issue, there is great controversy in the literature, and some authors describe that the reduction in the cervical implant portion could increase crestal bone loss [7, 8], while other authors argue that this can decrease the stresses on the bone crest by stabilizing the peri-implant tissues in this area [9, 10]. In addition, the increase in tissue volume in the cervical area of the implants causes a better sealing of this area, preventing the passage of bacteria and their produced fluids, thus avoiding possible complications and peri-implant diseases.

**Fig 1:**
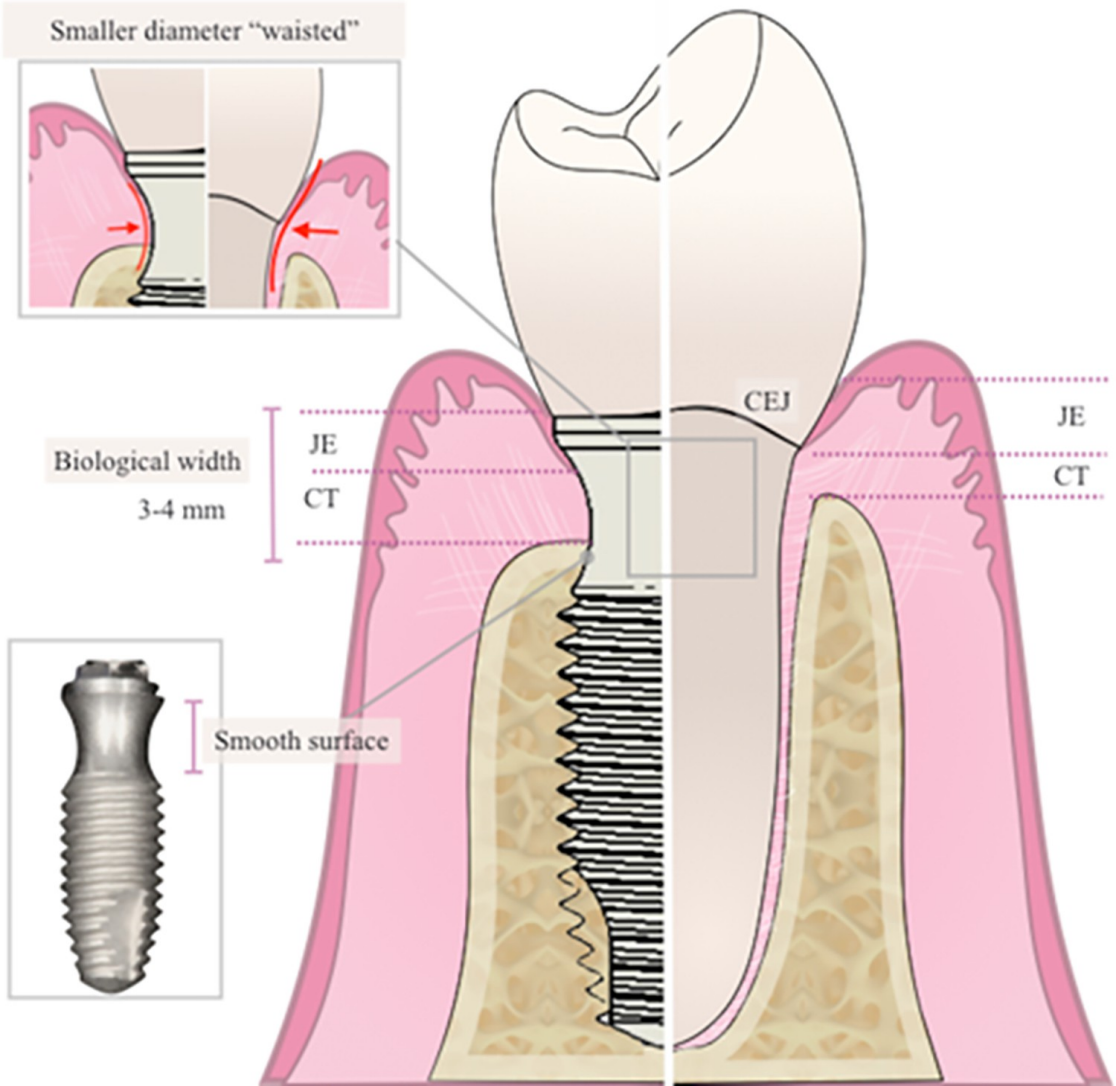
Comparison between the Collo implant and natural teeth design.

In this sense, a new implant design was proposed with the cervical portion presenting a design with concave and polished walls, thus increasing the amount of tissue in this area and, consequently, improving the health of the peri-implant tissues. A natural tooth has a narrower transition area between the root and the crown that can be seen as a neck, where the cementoenamel junction is located. This region harbors soft-tissue biological structures that make up the biological width (junctional epithelium and attached connective tissue) and adapt and promote the biological sealing of the intraosseous portion of the tooth root. Similarly, around dental implants, this barrier is essential to protect the underlying osseointegration from pathological external factors that may jeopardize soft and hard peri-implant tissues in a faster, accelerated, and unpredictable manner when compared to periodontitis [11], as demonstrated schematically in **Fig 1**.

However, like all new designs of implants or materials used for implantation, which will experience loads of different magnitudes, mechanical tests are essential to determine the supported levels without compromising their functionality. Thus, in the present study, we aimed to evaluate different limits of resistance of the new Collo implant in quasi-static test and in fatigue mechanical cycling, comparing it with two other conventional implant systems. The main hypothesis is that the new implant design presents limits of resistance values similar to conventional implants, regardless of the conditions tested (at bone level or outside of the bone).

## Materials and methods

**Fig 2:**
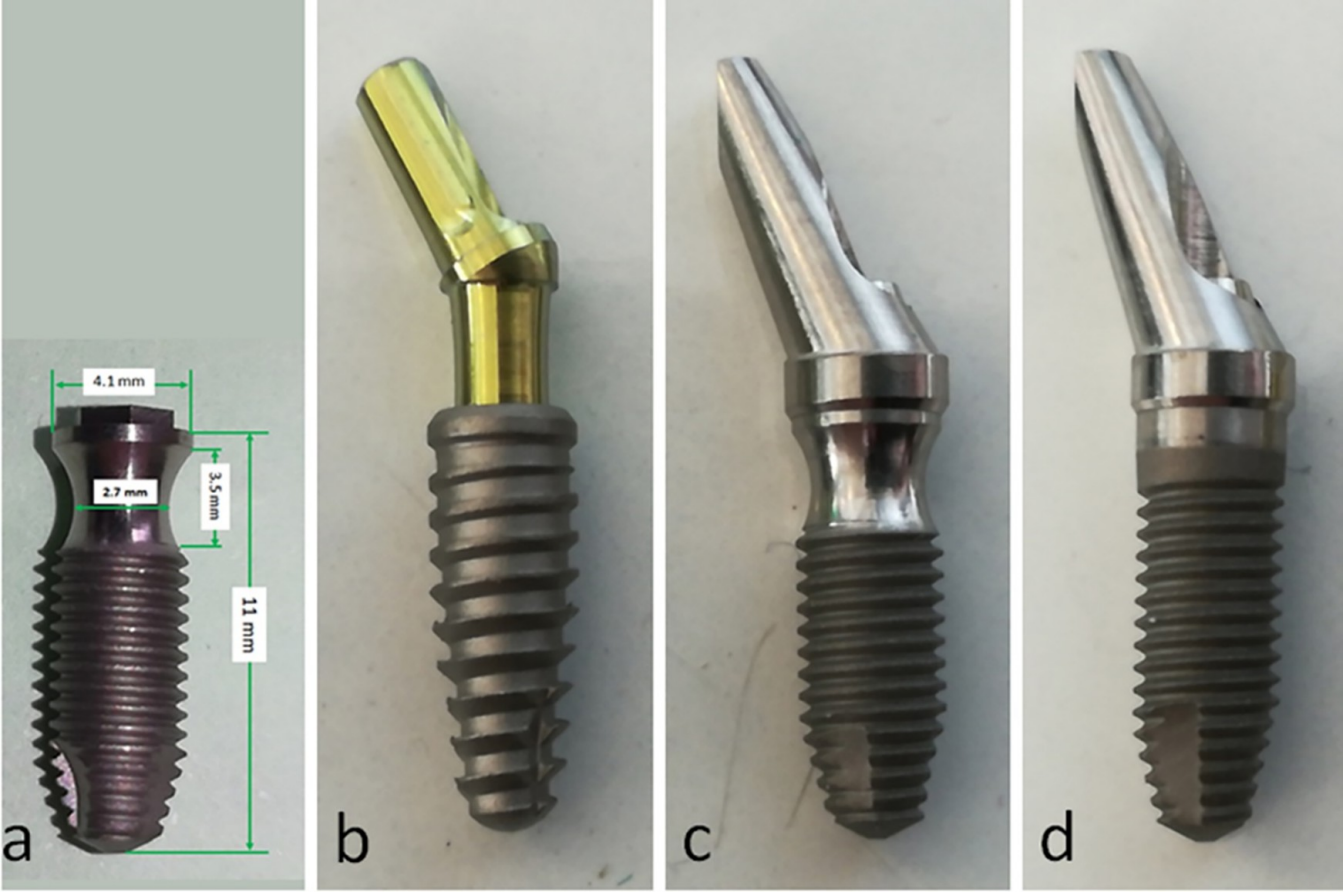
(a) Details of new Collo implant characteristics and dimensions; (b) sample implant and abutment used in the MT group; (c) sample implant and abutment used in the Collo out and Collo bl groups; and (d) sample implant and abutment used for the EH con group.

For the present study, 88 implants and 88 abutments were used, which were distributed into 4 groups (n = 22 per group): Morse taper connection implant (MT group), installed 2 mm infrabony according to the manufacturer’s recommendations; conventional external hexagon implant (EH con group), installed at bone level per the manufacturer’s recommendations; New Collo implant of external hexagon (Collo out group), installed with the polished part (referring to the implant neck) out of the bone tissue; New Collo implant of external hexagon (Collo bl group), with the implant platform inserted at the bone level. The inclusion of Morse taper connection implants in the present study was because this system has a concept of platform switching, presenting a reduction from the implant platform of 4 mm to 2.5 mm when the abutment is installed, in order to increase the volume of the tissues in this area. **Fig 2** shows the details about the new Collo implant dimensions and the three implant models used in the present study and their respective abutments used for the test. All the implants and abutments were manufactured by the company Implacil De Bortoli (São Paulo, Brazil). The implant dimensions used were of 11 mm in length and 4.1 mm in diameter for the Collo and EH groups, and 11 mm in length 4.0 mm in diameter for the MT group. All abutments were connected to the implants and received a torque of 30 Ncm.

### Quasi-static test

**Fig 3:**
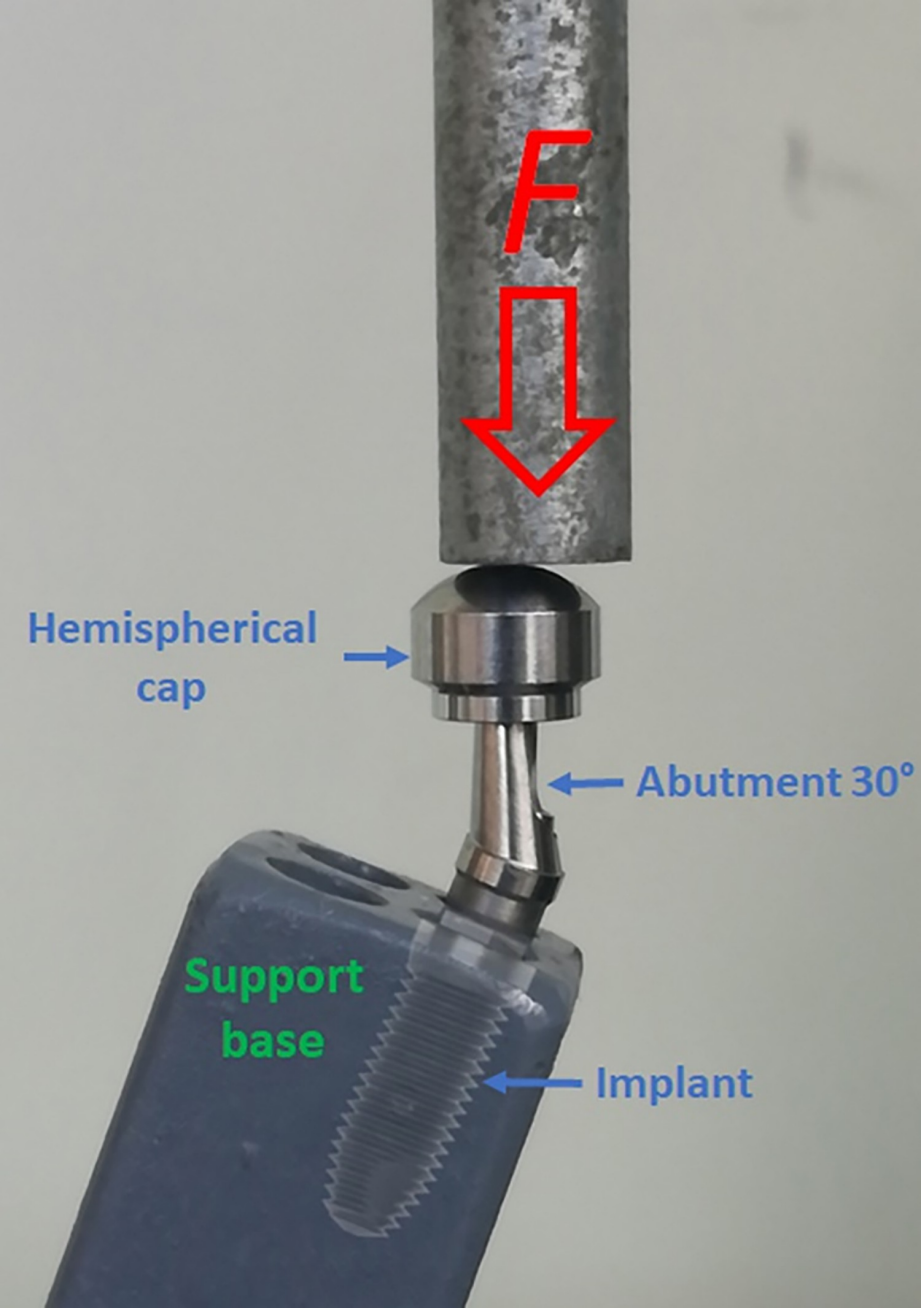
The components used in the test and the direction of load application on each sample.

Ten sets of each group were subjected to quasi-static loading in a universal testing machine AME-5kN (Oswaldo Filizola, São Paulo, Brazil), and the maximum resistance force supported by each sample was measured, generating a graph for each sample, which was analyzed later. The sample size was based on a power level of 85% to obtain a P value of .05, calculated by using a software program (SigmaStat 4.0; Systat Software Inc). For a desired power level of 85% with differences between the means and standard deviations of each group, the minimum sample size for each group under each condition was 8. Then, the implants were positioned into the test base with an inclination of 30 ± 1° in relation to the direction of the applied load. One abutment corresponding to each implant model with an angulation of 30° was selected, and over each abutment, a hemispherical cap was fabricated and accoupled to the abutment. These previously mentioned indications followed the specifications of the ISO 14801:2016 [12] standard for this type of test. **Fig 3** schematically shows the components used in the test and the direction of the load application on each sample.

**Fig 4:**
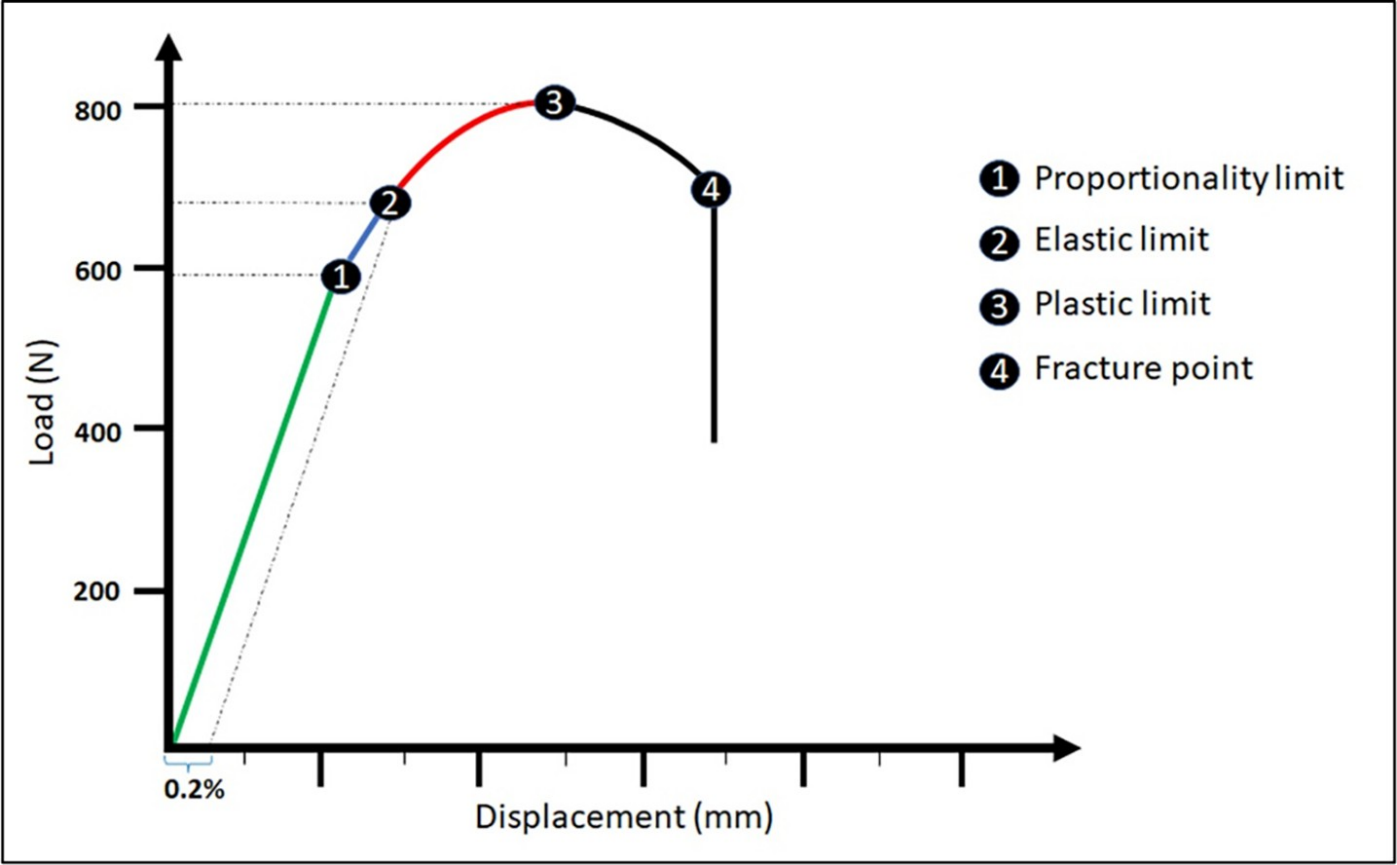
Graph demonstrating the limits of resistance that were evaluated in each set in each group.

Using the graphs generated in each test, the shape curve was evaluated during the application of the load on each sample, where the limits of proportionality, elastic, and plastic (maximum resistance) were analyzed, following the indications presented in the graph of **Fig 4**.

Point 4 of the graph in Fig 4, referring to the fracture point with the complete separation of the parts, was not evaluated because the test was stopped immediately after the curve reached the maximum limit of plastic deformation so that we could evaluate the damage to the samples up to that moment.

### Fatigue mechanical cycling test

The other 12 samples from each group were included in epoxy resin in the same conditions (implant shoulder position) used for the quasi-static test. Then, these samples were submitted to mechanical cycling in a mechanical cycler machine (BioPDI, São Carlos, Brazil), with the application of 2 x 10^6^ cycles with 4 different controlled axial force at a frequency of 2 Hz. Initially, a load of 80% of the maximum mean load value obtained in the quasi-static test in each group was used. Moreover, 3 others intensity of load were applied (60%, 40% and 20%). During mechanical cycling, the specimens were immersed in water at 37°C. Three samples were tested in each load intensity per group, in accordance with the ISO 14801 standard [12].

The statistical analysis was performed using the GraphPad Prism version 5.01 for Windows (GraphPad Software, San Diego, California, USA). All comparisons between the groups were performed by Bonferroni’s multiple comparison test, considering *p* < 0.05 as statistically significant.

## Results

### Quasi-static test results

**Fig 5:**
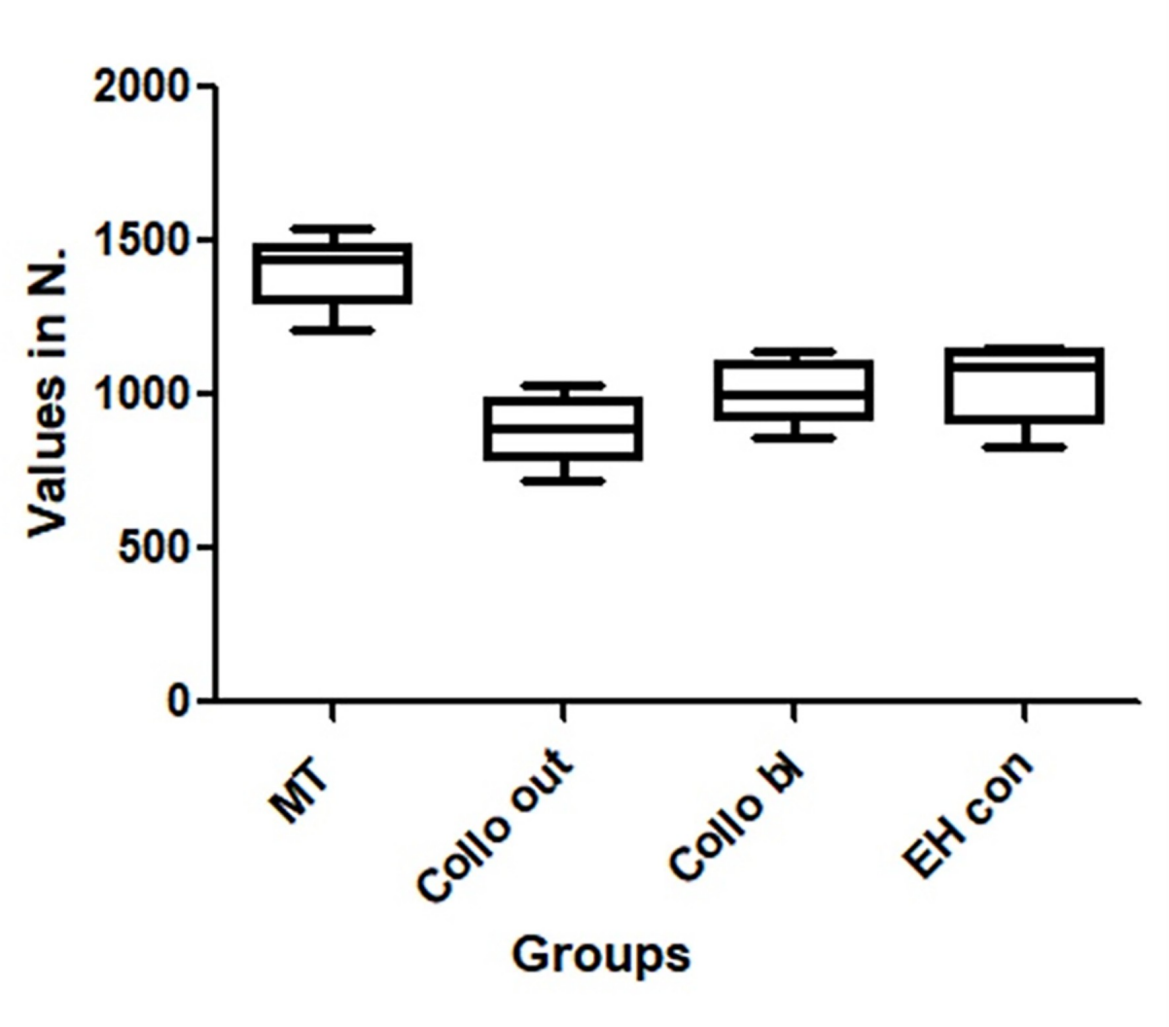
Image shows a box plot graph with the data distribution between the groups.

The mean and standard deviation (SD) of the maximum resistance to plastic deformation recorded during quasi-static loading of all the groups were 1397.6 ± 104.7 for the MT group, 889.4 ± 108.1 for the Collo out group, 1004.5 ± 96.7 for the Collo bl group, and 1046.5 ± 117.2 for the EH con group. **Fig 5** shows a box plot graph with the data distribution between the groups.

The EH con and Collo bl groups of implants showed similar maximum strength values of the sets (*p* > 0.05). The Collo out group showed the lowest resistance values in comparison to the other groups (*p* < 0.05). Meanwhile, samples from the MT group showed the highest resistance values in comparison to the other groups (*p* < 0.05). The detailed statistical comparison between the groups is presented in **Table 1**.

**Table 1:** Bonferroni’s multiple comparison test comparing the maximum resistance to plastic deformation between the groups.

Bonferroni’s Multiple Comparison Test	Mean difference	t	*p*-value	95% CI of difference
**MT vs Collo out**	508.2	10.63	< 0.0001 *	374.7 to 641.7
**MT vs Collo bl**	393.1	8.222	< 0.0001 *	259.6 to 526.6
**MT vs EH con**	354.1	7.406	< 0.0001 *	220.6 to 487.6
**Collo out vs Collo bl**	−115.1	2.407	0.0355 *	−248.6 to 18.38
**Collo out vs EH con**	−154.1	3.223	0.0068 *	−287.6 to −20.62
**Collo bl vs EH con**	−39.00	0.8157	0.4359	−172.5 to 94.48

*Statistically significant difference; CI = confidence interval.

**Fig 6:**
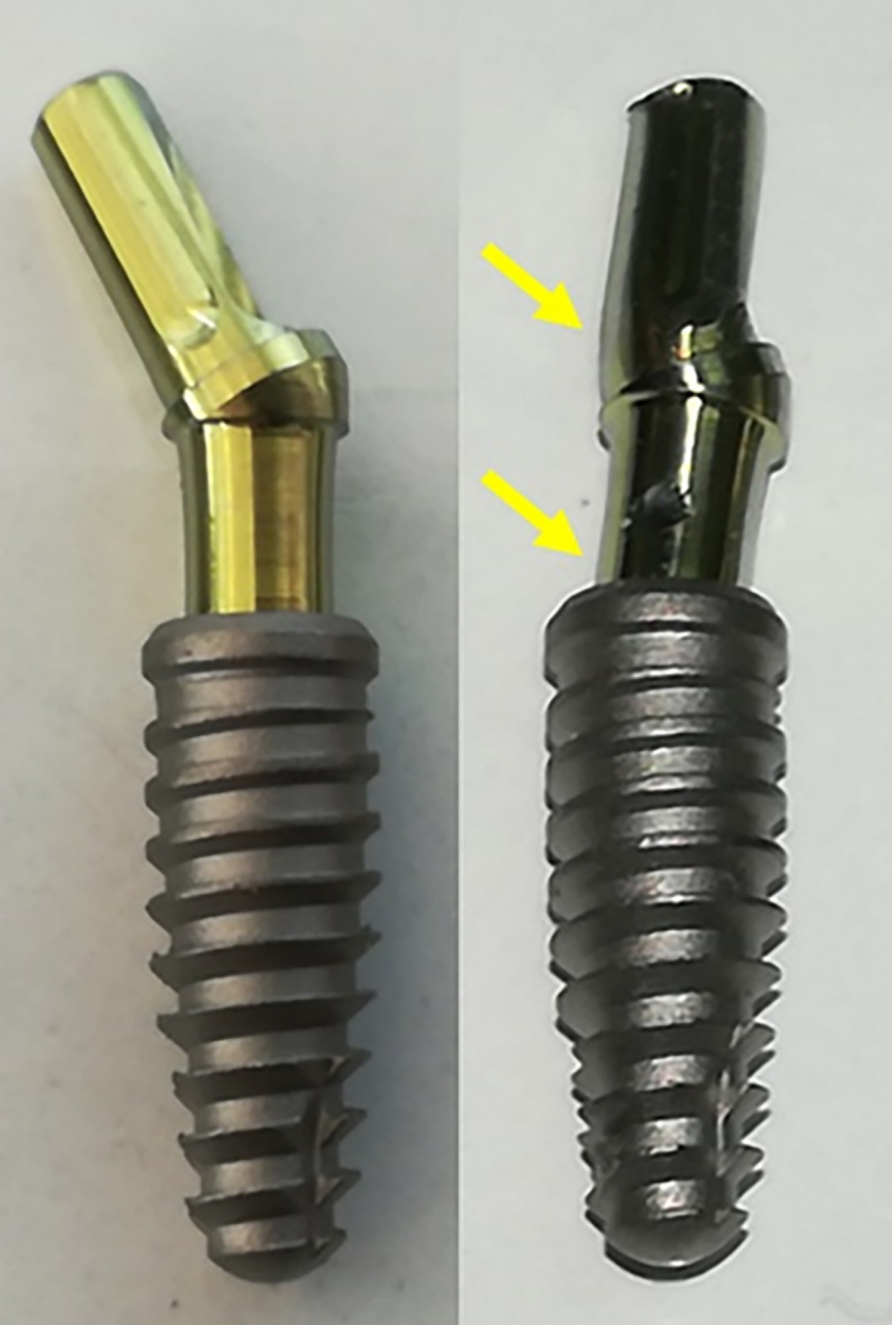
Representative images of a sample before (left) and after (right) the load application of the MT group. The yellow arrows indicate the points where the set showed less resistance.

By individually analyzing the images of the samples from each group, we can verify that in the MT group, there was a movement (bending) of the abutment in two positions, without presenting signals of fracture (**Fig 6**).

**Fig 7:**
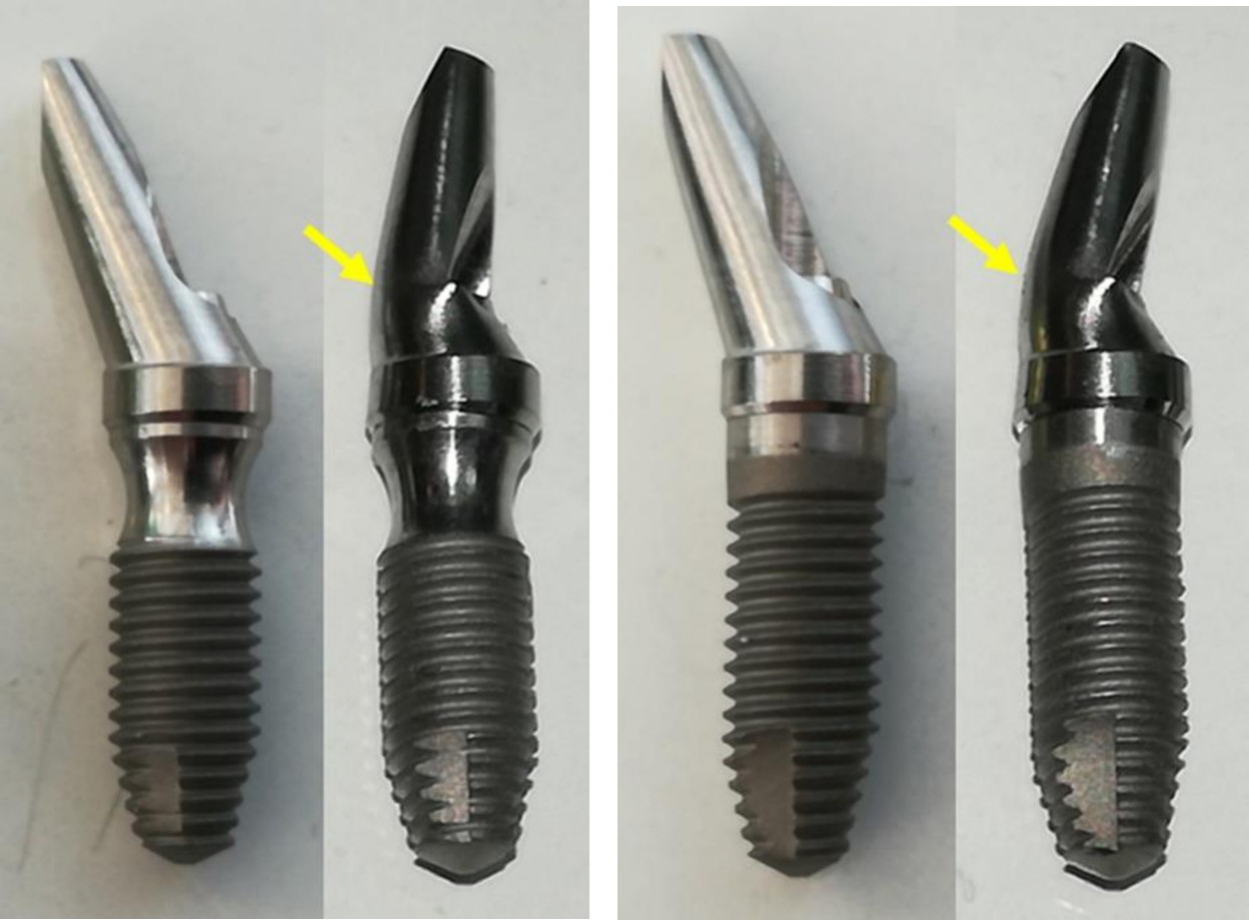
Representative images of a sample before (left) and after (right) the load application of the Collo bl group (a) and EH con group (b). The yellow arrows indicate the points where the set showed less resistance.

In the Collo bl and EH con groups, only bending of the abutment was observed in the same place in both groups, without showing signs of fracture (**Fig 7**).

**Fig 8:**
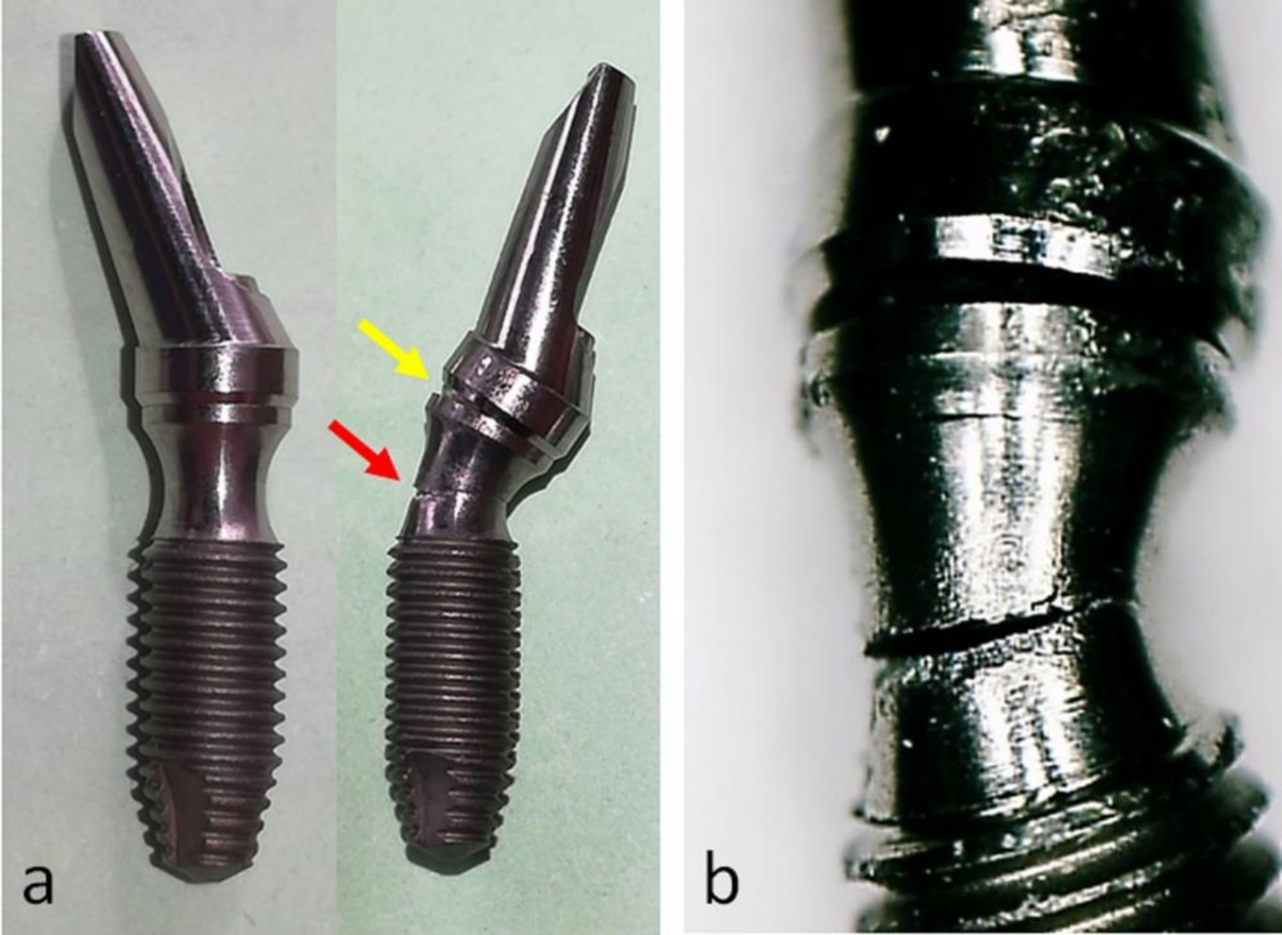
(a) Representative images of a sample before (left) and after (right) the load application of the Collo out group. The yellow arrow indicates where there was a separation between the abutment and implant, and the red arrow shows where the implant fracture occurred in its thinnest portion (center of the neck). (b) Image with higher magnification showing the details of the sample changes after the test.

However, the Collo out group, where the polished part of the cervical portion (neck) of the implant was outside the insertion, presented a fracture in this portion of the implant and displacement of the abutment on the implant platform (**Fig 8**).

The mean and SD of the proportionality limit found from analyzing the graphs after the test of all groups were 1094.4 ± 97.3 N for the MT group, 711.2 ± 91.8 N for the Collo out group, 780.8 ± 89.8 N for the Collo bl group, and 794.3 ± 97.1 N for the EH con group. The mean and SD of the elastic limit analyzed in the graphs for all groups were 1248.2 ± 92.1 N for the MT group, 791.3 ± 87.6 N for the Collo out group, 881.8 ± 90.1 N for the Collo bl group, and 913.9 ± 101.1 N for the EH con group. The statistical analysis between the groups showed the same relationships presented in the comparisons between the groups of the values obtained for the maximum resistance to plastic deformation.

### Fatigue mechanical cycling test results

**Fig 9:**
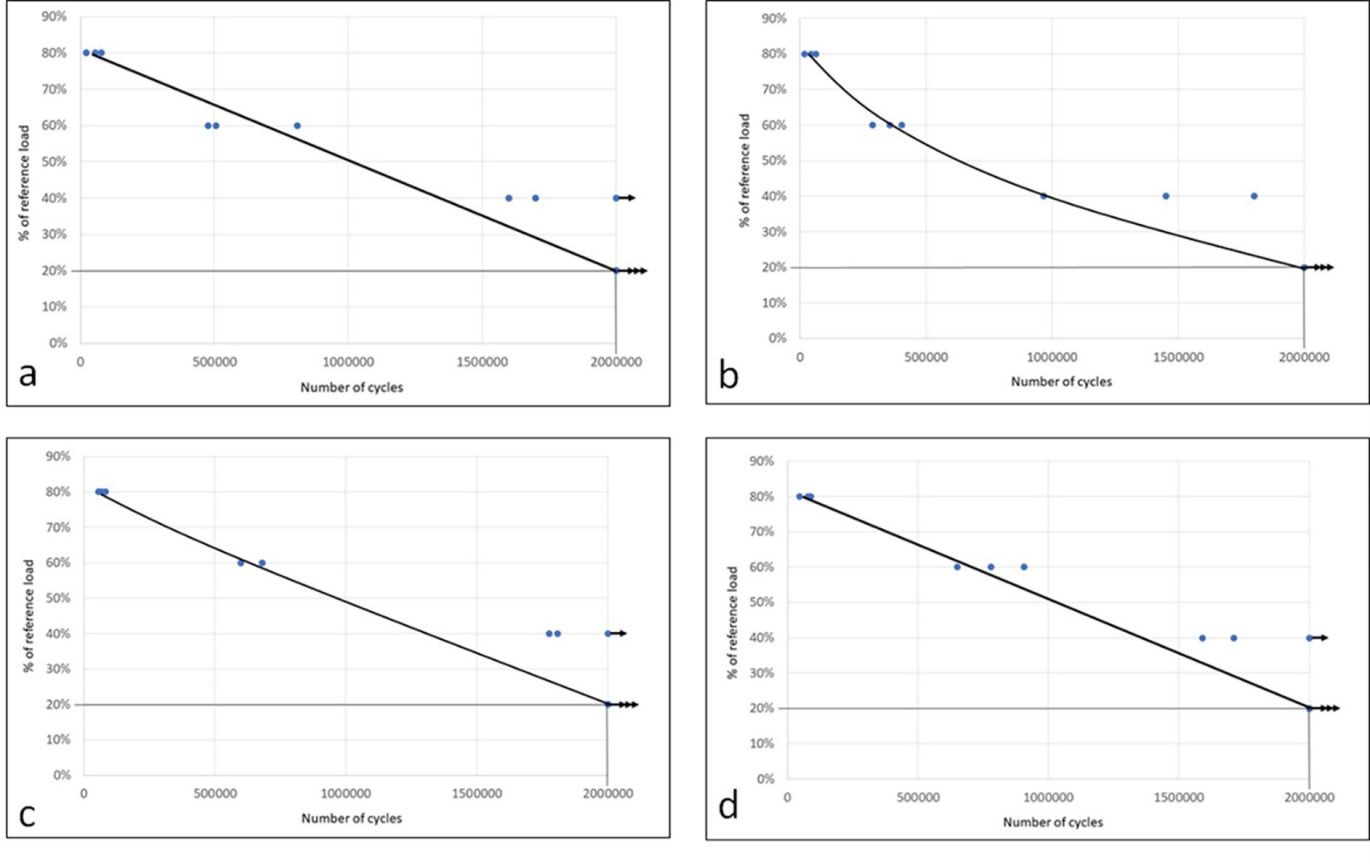
Graph images of the number of cycles supported by the samples of each group during the 4 applied load levels: (a) MT group, (b) Collo out group, (c) Collo bl group, and (d) EH con group.

**Table 2** presents the results with the averages of cycles supported by the samples of each group in the 4 intensities of forces calculated on the value of maximum resistance obtained in the quasi-static test, as well as the statistical comparison (ANOVA test). In **Fig 9** the graphs show the distribution of samples from each group after the cyclic fatigue test. The Collo out group had a lower average number of cycles compared to the other 3 groups at 3 load levels (80%, 60%, and 40%). However, at the load value of 20% all groups had the same performance.

**Table 2:** Mean and statistical comparison of cycles supported by the samples of each group in the 4 intensities of forces calculated on the value of maximum resistance obtained in the quasi-static test.

Load Groups	80%	60%	40%	20%
MT	51,000	596,667	1,766,667	2,000,000
Collo out	41,333	349,667	1,405,667	2,000,000
Collo bl	68,000	652,500	1,861,667	2,000,000
HE con	70,233	779,196	1,766,667	2,000,000
ANOVA test	0.3919	0.0125*	0.2332	--

*Statistically significant difference.

## Discussion

The re-establishment of the peri-implant “biological width” may lead to undesirable marginal bone loss, known as saucerization. Based on these findings, modifications in the coronal part of the implants and in the prosthetic abutment have been made to suit the structures of the biological space, thus avoiding saucerization. The modification of the new Collo implant is based on earlier evidence that demonstrates that 3–4 mm is needed for the junctional epithelium and the connective adaptation, promoting a similar biological sealing that occurs in natural teeth [8, 13]. However, the evaluation of the behavior of implantable materials prior to their use in humans entails *in vitro* tests. These tests are usually guided by international standards, which determine the conditions and dictate some requirements for these materials to be registered and commercialized. Fatigue tests that simulate in vivo conditions are commonly accepted to provide data on longevity and fracture resistance of implants [14–16]. These tests are duly recognized by the International Organization for Standardization through ISO 14801 [11]. In the present study, we performed the quasi-static fatigue test until the specimen reached the maximum strength limit. Thus, the null hypothesis tested—which assumed that, regardless of the conditions, the different implant designs would present similar mechanical performance in terms of resistance to loads—was not confirmed by the results found.

The mechanical strength between the sets (abutment and implant) is of fundamental importance for the long-term success of this type of rehabilitation, through the allowance of short movements at the joint interface (presence of gaps) and flexural fatigue of the materials. According to Santos et al. [17], the design and material used in the confection of the sets clearly influence the processes of plastic deformation, wear, or failure of these parts. In the present study, different designs of implants and abutments were tested, all fabricated with the same material. The selection of implant models to be used took into account the type of connection of the new Collo implant, so it was compared with a conventional external hexagon implant and the characteristics of the Morse taper implant system that features the abutment with a reduced neck in relation to the diameter of the implant. In addition, regarding the implant with Morse taper connection, the authors demonstrated that these implants exhibit better results in terms of abutment fit, stability, and seal performance [18, 19], in addition to presenting a better distribution of loads on the peri-implant bone tissue [20]. In another comparative study of compressive forces between different internal connection and external hexagon implants, we found that Morse taper sets (implant and abutment) provided resistance to compressive loading [21, 22], corroborating the results found in the present study.

The use of an angled component is exclusively used in specific clinical situations, in which the implant was installed outside the ideal axial situation. Therefore, the regulation itself provides for analysis using the situation considered most critical found in the clinical use of the implant [12]. Analyzing the images of the samples after the test, all of them showed failures in the thinner portion of the sets, which in the MT, Collo bl, and EH groups was in the abutment, while in the Collo out group, the greater loss of resistance was in the reduced portion of these implants (neck).

During masticatory movements, most of the applied forces are vertical, that is, compressive forces. Therefore, the compressive strength of dental implants and abutments is a subject that has been extensively researched and discussed in the literature [14, 15, 23, 24]. However, although there is an established standard for this type of quasi-static or cyclic compressive strength test (ISO 14801) [12], modifications in the tests are adapted according to the object of each research investigation. In our study, differently from what the aforementioned standard recommends, the implants were positioned at different levels and, in relation to the support base, simulated an ideal situation for each implant model, mainly to verify if the new Collo implant could be installed with its reduced portion (neck) out of the bone tissue without affecting the strength values. The results showed lower resistance values for the neck implant installed outside the bone, which could probably occur with the other models tested, as demonstrated in previous studies that show that, regardless of the implant model, when it has bone attachment loss crestal, there will be a decrease in resistance [21, 22]. However, the average presented by the Collo out group (889.4 N) was higher than the values of maximum occlusal force reported in the literature, which would be between 180 N and 850 N in the first molar region, and values between 95 N and 250 N in the incisors [25, 26].

The fatigue loading frequency established by ISO14801 [12] is 15 Hz for dry air, however, the human chewing frequency ranges from 1 to 4 Hz [27]. The high frequencies can favor the characteristics of the material, but they do not reproduce the human condition. Thus, loads should be limited to 2 Hz, simulating an accumulation of clinically relevant damage [28]. For this reason, a frequency of 2 Hz was used in the present study. The results of the fatigue test showed that the Collo out group supported a smaller number of cycles that led to the fracture than the other 3 groups proposed at loads of 80%, 60%, and 40%, and only at the load value of 20% all groups had the same performance. However, due to the small number of samples tested at each load value, the statistical analysis did not detect a difference between the groups for loads of 80% and 40%, only detected difference for load at 60%. However, the Collo out group had a number of cycles approximately 20% lower in comparison with the other group that presented the lower values in each of these 2 loads (80% and 40%).

Few studies are found in the literature analyzing the limits of proportionality, elastic, and plastic (maximum resistance) of implant and abutment sets. Recently, authors reported that the knowledge of mechanical properties can help dentists choose the correct materials, since comparisons were made between old and new designs and/or projects, as well as comparisons with leading brands highlighted in the world market [29]. Thus, we sought to highlight these resistance limits in the tested sets, and the values showed that, in relation to the plastic limit (maximum resistance), the elastic limit detected was, on average, 10% lower, and the proportionality limit presented values on average 20% smaller.

Still, it is important to emphasize that *in vitro* assays contribute to the study of the mechanical behavior of implantable materials, but they are not necessarily representative of the results of clinical follow-ups due to their limitations—that is, the tests do not simulate the physiological behavior (variations of temperature, presence of oral fluid, occlusal interference, conditions of bruxism, among others) or the dynamic muscular action typical of the muscles of mastication. On the other hand, the stiffness of the support base (metallic) is different from the density of the bone tissue, causing different stress distribution; therefore, they do not simulate the interrelationship between implant and bone. It is also important to emphasize the number of samples that are always tested in *in vitro* studies, which, despite being adequate by the power calculation, is practically insignificant given the amount of parts manufactured and marketed worldwide. Future studies and projects should be carried out to try to improve these failures and minimize them so patients will not incur negative and costly consequences. Also, in silico finite element analysis will be helpful to predict crack line after the fatigue test [30].

## Conclusions

Within the limitations of the present *in vitro* study, it was possible to conclude that the new Collo implant, regardless of the bone level, presented resistance values within acceptable levels for use as a support for crowns for the replacement of lost teeth. However, better mechanical performance of the new Collo implant was observed when it was installed at the bone level.

## References

[1] Wang Y, Zhang Y, Miron RJ. Health, Maintenance, and Recovery of Soft Tissues around Implants. Clin Implant Dent Relat Res. 2016;18(3):618–34. doi: 10.1111/cid.12343 25873299

[2] Monje A, Aranda L, Diaz KT, Alarcón MA, Bagramian RA, Wang HL, Catena A. Impact of Maintenance Therapy for the Prevention of Peri-implant Diseases: A Systematic Review and Meta-analysis. J Dent Res. 2016;95(4):372–9. doi: 10.1177/0022034515622432 26701350

[3] Rösing CK, Fiorini T, Haas AN, Muniz FWMG, Oppermann RV, Susin C. The impact of maintenance on peri-implant health. Braz Oral Res. 2019;33(suppl 1):e074. doi: 10.1590/1807-3107bor-2019.vol33.0074 31576958

[4] Linkevicius T, Apse P, Grybauskas S, Puisys A. The influence of soft tissue thickness on crestal bone changes around implants: a 1-year prospective controlled clinical trial. Int J Oral Maxillofac Implants. 2009;24(4):712–9. 19885413

[5] Gharpure AS, Latimer JM, Aljofi FE, Kahng JH, Daubert DM. Role of thin gingival phenotype and inadequate keratinized mucosa width (<2 mm) as risk indicators for peri-implantitis and peri-implant mucositis. J Periodontol. 2021;92(12):1687–1696. 33856690 10.1002/JPER.20-0792

[6] Thoma DS, Gil A, Hämmerle CHF, Jung RE. Management and prevention of soft tissue complications in implant dentistry. Periodontol 2000. 2022;88(1):116–129. doi: 10.1111/prd.12415 35103320 PMC9306802

[7] Carinci F, Brunelli G, Danza M. Platform switching and bone platform switching. J Oral Implantol. 2009;35(5):245–50. doi: 10.1563/AAID-JOI-D-09-00022.1 19882821

[8] Messias A, Nicolau P, Guerra F. Titanium dental implants with different collar design and surface modifications: A systematic review on survival rates and marginal bone levels. Clin Oral Implants Res. 2019;30(1):20–48. doi: 10.1111/clr.13389 30466192

[9] Tan WC, Lang NP, Schmidlin K, Zwahlen M, Pjetursson BE. The effect of different implant neck configurations on soft and hard tissue healing: a randomized-controlled clinical trial. Clin Oral Implants Res. 2011;22(1):14–9. doi: 10.1111/j.1600-0501.2010.01982.x 21091792

[10] Dávila E, Ortiz-Hernández M, Perez RA, Herrero-Climent M, Cerrolaza M, Gil FJ. Crestal module design optimization of dental implants: finite element analysis and in vivo studies. J Mater Sci Mater Med. 2019;30(8):90. doi: 10.1007/s10856-019-6291-1 31346767

[11] Sukekava F, Pannuti CM, Lima LA, Tormena M, Araújo MG. Dynamics of soft tissue healing at implants and teeth: a study in a dog model. Clin Oral Implants Res. 2016;27(5):545–52. doi: 10.1111/clr.12621 26031414

[12] International Organization for Standardization (2007) ISO 14801: dentistry-implants-dynamic fatigue test for endosseous dental implants. The Organization, Geneva, Switzerland.

[13] Gracis S, Llobell A, Bichacho N, Jahangiri L, Ferencz JL. The Influence of Implant Neck Features and Abutment Diameter on Hard and Soft Tissues Around Single Implants Placed in Healed Ridges: Clinical Criteria for Selection. Int J Periodontics Restorative Dent. 2020;40(1):39–48. doi: 10.11607/prd.4151 31815971

[14] Quek HC, Tan KB, Nicholls JI. Load fatigue performance of four implant-abutment interface designs: effect of torque level and implant system. Int J Oral Maxillofac Implants. 2008;23(2):253–62. 18548921

[15] Sailer I, Sailer T, Stawarczyk B, Jung RE, Hämmerle CH. In vitro study of the influence of the type of connection on the fracture load of zirconia abutments with internal and external implant-abutment connections. Int J Oral Maxillofac Implants. 2009;24(5):850–8. 19865625

[16] Dittmer MP, Dittmer S, Borchers L, Kohorst P, Stiesch M. Influence of the interface design on the yield force of the implant-abutment complex before and after cyclic mechanical loading. J Prosthodont Res. 2012;56(1):19–24. doi: 10.1016/j.jpor.2011.02.002 21398198

[17] Santos MD, Pfeifer AB, Silva MR, Sendyk CL, Sendyk WR. Fracture of abutment screw supporting a cemented implant-retained prosthesis with external hexagon connection: a case report with sem evaluation. J Appl Oral Sci. 2007;15(2):148–51. doi: 10.1590/s1678-77572007000200015 19089120 PMC4327248

[18] Gehrke SA, Delgado-Ruiz RA, Prados Frutos JC, Prados-Privado M, Dedavid BA, Granero Marín JM, et al. Misfit of Three Different Implant-Abutment Connections Before and After Cyclic Load Application: An In Vitro Study. Int J Oral Maxillofac Implants. 2017;32(4):822–829. doi: 10.11607/jomi.5629 28708914

[19] Gehrke SA, Dedavid BA, Marín JMG, Canullo L. Behavior of implant and abutment sets of three different connections during the non-axial load application: An in vitro experimental study using a radiographic method. Biomed Mater Eng. 2022;33(2):101–112. doi: 10.3233/BME-211221 34511480

[20] Zanatta LC, Dib LL, Gehrke SA. Photoelastic stress analysis surrounding different implant designs under simulated static loading. J Craniofac Surg. 2014;25(3):1068–71. doi: 10.1097/SCS.0000000000000829 24777027

[21] Gehrke SA, Souza Dos Santos Vianna M, Dedavid BA. Influence of bone insertion level of the implant on the fracture strength of different connection designs: an in vitro study. Clin Oral Investig. 2014;18(3):715–20. doi: 10.1007/s00784-013-1039-7 23860902

[22] Prados-Privado M, Gehrke SA, Rojo R, Prados-Frutos JC. Probability of Failure of Internal Hexagon and Morse Taper Implants with Different Bone Levels: A Mechanical Test and Probabilistic Fatigue. Int J Oral Maxillofac Implants. 2018;33(6):1266–1273. doi: 10.11607/jomi.6426 30427957

[23] Cibirka RM, Nelson SK, Lang BR, Rueggeberg FA. Examination of the implant-abutment interface after fatigue testing. J Prosthet Dent. 2001;85(3):268–75. doi: 10.1067/mpr.2001.114266 11264934

[24] Dittmer MP, Dittmer S, Borchers L, Kohorst P, Stiesch M. Influence of the interface design on the yield force of the implant-abutmentcomplex before and after cyclic mechanical loading. J Prosthodont Res. 2012;56(1):19–24. doi: 10.1016/j.jpor.2011.02.002 21398198

[25] Haraldson T, Carlsson GE, Ingervall B. Functional state, bite force and postural muscle activity in patients with osseointegrated oral implant bridges. Acta Odontol Scand. 1979;37(4):195–206. doi: 10.3109/00016357909027582 291276

[26] Paphangkorakit J, Osborn JW. The effect of pressure on a maximum incisal bite force in man. Arch Oral Biol. 1997;42(1):11–7. doi: 10.1016/s0003-9969(96)00106-9 9134111

[27] Sakaguchi RL, Douglas WH, DeLong R, Pintado MR. The wear of a posterior composite in an artificial mouth: a clinical correlation. Dent Mater. 1986;2(6):235–40. doi: 10.1016/s0109-5641(86)80034-3 3468027

[28] Karl M, Kelly JR. Influence of loading frequency on implant failure under cyclic fatigue conditions. Dent Mater. 2009;25(11):1426–32. doi: 10.1016/j.dental.2009.06.015 19643468

[29] Vaidya A, Pathak K. Chapter 17: Mechanical stability of dental materials, Editor(s): Asiri AM, Ali Mohammad I. In Woodhead Publishing Series in Biomaterials, Applications of Nanocomposite Materials in Dentistry. Woodhead Publishing, 2019, Pag. 285–305. ISBN 9780128137420.

[30] Yamaguchi S, Yamanishi Y, Machado LS, Matsumoto S, Tovar N, Coelho PG, et al. In vitro fatigue tests and in silico finite element analysis of dental implants with different fixture/abutment joint types using computer-aided design models. J Prosthodont Res. 2018;62(1):24–30. doi: 10.1016/j.jpor.2017.03.006 28427837

